# A Proposed Nursing Service for the Territorial Army

**Published:** 1908-06-27

**Authors:** 


### The Royal Army Medical Corps Section.

### A PROPOSED NURSING SERVICE FOR THE TERRITORIAL ARMY.

When a general hospital of the Territorial Army is fully mobilised its complete staff numbers 221. Of this total 10 are male ward orderlies and 91 are female nurses, the ranks of the latter including one matron, 22 sisters, and 68 staff nurses. As there are 23 general hospitals in the United Kingdom this gives a total of 23 matrons, 506 sisters, and 1,564 staff nurses required for the nursing needs of these hospitals, but with the view of easing liability for duty and to suit the special conditions of the nursing profession in civil life, it has been decided that the number of nurses to be borne on the roll of each general hospital will be 120, made up of two matrons, 30 sisters, and 88 staff nurses, so that the total number of nurses needed will be 2,760, of whom 46 will be matrons, 690 sisters, and 2,024 staff nurses.

How to provide this nursing staff is the question at present engaging the attention of the military authorities, and their idea is to form a Territorial Nursing Service similar to that of the Regular Army. With this object they have held conferences with the matrons of several of the largest hospitals in London, Edinburgh, Glasgow, Manchester, and other places, and everywhere the project has been favourably received. Nothing has been yet definitely done as to the rules and regulations for this Nursing Service, but they will certainly embody the following principles:—(1) A nurse may resign at any time; (2) the rate of pay and allowances will be those given to matrons, sisters, and staff nurses of the Army Nursing Service Reserve. These are as follows:—

| | Initial Rate £ | Annual Increment £ s. | Maximum £ |
|---|---|---|---|
| Matron ... ... ... ... | 75 ... | 10 0 ... | 150 |
| Sister ... ... ... ... | 50 ... | 5 0 ... | 65 |
| Staff Nurse ... ... ... | 40 ... | 2 10 ... | 45 |

(3) There will be a system of gratuities, which will be at the rates of £15 for matrons, £10 for sisters, and £7 10s. for staff nurses. All members of the Territorial Nursing Service doing duty in a general hospital will receive the above gratuities on the cessation of their employment, provided they are certified by the principal medical officer under whom they have served to have rendered satisfactory service. If, however, a nurse has relinquished her employment for reasons not satisfactory to the Army authorities, she will forfeit her title to the gratuity. On the other hand, when a nurse's employment has extended beyond one year she will be granted, under the same conditions and at the same rates, a further gratuity for a complete year of service, broken periods to be calculated accordingly. (4) Certain allowances will also be granted, of which the following are the chief: (*a*) In lieu of board and washing, 15s. per week; (*b*) for clothing and cloak, £9 a year; (*c*) Government quarters, with fuel and light; (*d*) travelling expenses from the place of residence to the general hospital when called up for duty.

The organisation by which it is proposed to establish and work this nursing service is to consist of (1) a Central Council, and (2) small Local Committees. The Central Council has just been appointed by the War Office, and numbers 14. It is composed of a chairman with six lady members and seven hospital matrons. To this Council will fall the duties of deciding on the uniform to be worn and of drawing up the qualifications and rules of the Service, basing the latter, as far as possible, upon those in force in the Regular Army and in accordance with the principles already enumerated. The Local Committees will be formed at the cities or towns where the general hospitals are located. They will be nominated by the Central Council, and each one will include an organising matron. It is they who will enroll the names of nurses qualified and willing to serve on the staff of the local general hospital, and they will receive applicants up to the number of 120. Candidates should be taken preferably from the area in which their general hospital is situated, although the names of nurses outside the area will also be accepted.

By such an organisation as this it is evident that the military authorities favour the system of enrolling in time of peace nurses individually by name rather than that of entering into agreements with the various hospitals or training schools in the United Kingdom to furnish a certain number of nurses on the outbreak of war or on the mobilisation of the Territorial Force. The reasons that have influenced them in favour of their system are that under it they will have rapidity of mobilisation and a certainty of their reserves. A little consideration will, however, show that this idea is not altogether correct, and that with it are associated some of those very drawbacks that it is desirable to avoid. In the first place, the nurses obtained under it will be from the ranks of those who have left the hospitals and are engaged in private work, for matrons of hospitals would naturally not favour the retention on their staff of nurses who have an allegiance to an outside body, especially if a retaining fee is given, as has been suggested, and if they are liable to be called away at any time to an annual training, as has also been proposed. Now it is very generally admitted that the conditions of the nurse's vocation in private work are characterised by uncertainty and rapidity of change, so that no sooner has she joined the Territorial Nursing Service than she may find herself so placed that she cannot fulfil her obligation except to the detriment of some patient or of some other appointment she has taken up. Another very important point, too, is that the plan of registering individual names cannot ensure the quality of the nurses obtained. It is not necessary to do more than mention what all matrons know, that many nurses are not adapted to the work and surroundings of a military hospital, and yet by the plan of individual registration selection would practically be non-existent; for no local committee can form an opinion as to the existence in a candidate of those personal qualifications which are as necessary in this case as nursing ability and skill.

We would rather favour the idea of entering beforehand into an agreement with the large hospitals in the United Kingdom to furnish a certain number of nurses on the outbreak of war or on the mobilisation of the Territorial Army. We believe that the matrons of all these hospitals would willingly agree to keep a roster of suitable nurses up to a certain number. By such an arrangement as this we think that a reliable, inexpensive, and more acceptable form of organisation would be secured for the supply of nurses to the Territorial General Hospitals, while by it the quality of the nurses would be of a high order and to some extent guaranteed. It may be taken for granted that in time of invasion the civil hospitals would be proud to put up with any temporary inconveniences, and would send their best and most suitable nurses to the general hospitals, while the nurses, on their part, would not only be eager to nurse the sick and wounded, but would be actuated by a desire to keep up the credit of their respective hospitals. After a careful consideration of the whole matter we are of opinion that the organisation proposed by the military authorities is unsuitable and will have a tendency to lessen the interest of the large hospitals in this new nursing service. This would be an unfortunate result, and it will be in every way better to rely on the resources of these institutions than to insist on a plan that would have indirectly a tendency to alienate them from it.

